# A Methodology for Validating Diversity in Synthetic Time Series Generation^☆^

**DOI:** 10.1016/j.mex.2021.101459

**Published:** 2021-07-24

**Authors:** Fouad Bahrpeyma, Mark Roantree, Paolo Cappellari, Michael Scriney, Andrew McCarren

**Affiliations:** a Insight Centre for Data Analytics, School of Computing, Dublin City University, Dublin 9, Ireland; b City University of New York, 2800 Victory Blvd, Staten Island, 10314 NY, USA; c VistaMilk SFI Research Centre, Dublin City University, Dublin 9, Ireland

**Keywords:** Synthetic time series, Time series features, Diversity, Coverage, Forecasting

## Abstract

In order for researchers to deliver robust evaluations of time series models, it often requires high volumes of data to ensure the appropriate level of rigor in testing. However, for many researchers, the lack of time series presents a barrier to a deeper evaluation. While researchers have developed and used synthetic datasets, the development of this data requires a methodological approach to testing the entire dataset against a set of metrics which capture the diversity of the dataset. Unless researchers are confident that their test datasets display a broad set of time series characteristics, it may favor one type of predictive model over another. This can have the effect of undermining the evaluation of new predictive methods. In this paper, we present a new approach to generating and evaluating a high number of time series data. The construction algorithm and validation framework are described in detail, together with an analysis of the level of diversity present in the synthetic dataset.

## Highlights

• This paper presents a new method for generating 50K diverse synthetic time series.
• We present a discussion on time series characteristics and metrics with a view to understanding time series diversity.
• We developed a robust framework for validating diversity in synthetic time series generation.

## Graphical abstract

**Figure:**
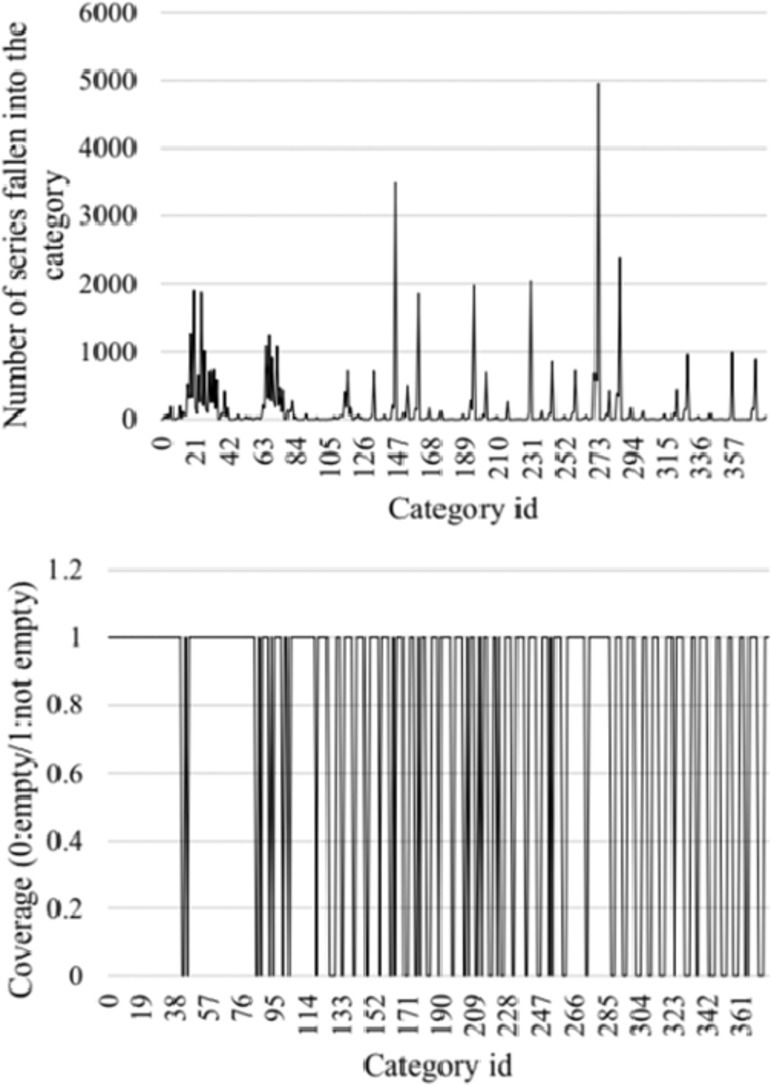

Specifications table

**Table:** 

Subject area:	Data Science
More specific subject area:	Time Series
Method name:	A Methodology for Validating Diversity in Synthetic Time Series Generation
Name and reference of original method:	N.A
Resource availability:	Method & Datasets available on Zenodo

## Introduction

Time series analysis has long been an interesting topic of research across multiple domains, as many systems require a sequential monitoring of their data streams at constant intervals. Daily prices, weekly stock indices, hourly temperature and monthly precipitation rates are examples of the domains where a sequential monitoring is incorporated. There are currently multiple challenges in time series analysis such has time series classification, time series clustering, feature learning and time series prediction. Time series classification is a process employed to label unseen time series into a set of pre-existing classes of time series [1]. Time series classification has applications in various domains such as EEG signal analysis and fault detection [1]. Feature learning is the process of extracting and learning features from time series data in order to improve time series classification. Many studies have been conducted in feature learning such as [2] and [3] which generate discriminative time series features in an attempt to improve the performance of time series classification (Table 1).

Our previous studies in time series prediction [4] suggest that evaluating new methods for time series predictions generally takes place using datasets that are specific to a researcher’s area of interest. The acquisition of data involves harvesting from specific studies or bodies that have an interest in a particular research question. However, this can mean that solutions are not applicable to other domains. There are numerous competition and open source datasets repositories that have been made available to the research community, such as BCI [5] and Kaggle [6]. These datasets have allowed researchers to test their methods more extensively, but the datasets are not typically classified by *time series* evaluation metrics but by *accuracy* of the methods employed.

The primary issue for time series researchers is the lack of available data to ensure robust validation. Synthetic data has been mixed with real life data in an attempt to broaden the scope of existing methods but the range of time series characteristics is quite narrow and datasets can be relatively small. There are many examples of researchers generating synthetic time series, such as: synthetic data generation [7]; surrogate data analysis [8]; using heuristics to materialize datasets [9]; and simulated data [10]. Surrogate data analysis can be used to estimate the impact of the *scale* of a time series characteristic through the comparison of the given time series with surrogate series [8]. This can then be used to estimate the impact of non-linearity in a time series in comparison to a series generated from a linear models such as ARIMA [11] and thus, allows researchers to replicate statistical features such as auto-correlation.

Time series clustering is a type of analysis that identifies similar time series and places them into a set of distinct groups. Time series clustering has applications in domains such as community detection and social media analysis. Studies such as [12] present new features known as *shapelets* to improve time series clustering. Shapelets are useful as features for classification and clustering but as it is not a statistical time series feature, it cannot be measured quantitatively to demonstrate diversity and coverage. Other approaches included [7], where the authors used a Markov chain model to create synthetic time series and an approach synthetic data creation for specific types of time series such as critical transitions [13]. In [14], a stochastic approach was presented to reproduce *long range* persistence of time series at multiple scales. An approach called Generative Adversarial Networks (GAN) [15] adopted machine learning algorithms to generate time series.

**Problem Definition.** Time series data generally exhibits a number of key characteristics (described later) and an important consideration when generating synthetic data is for these properties to occur with a fairly even level of distribution. We refer to this as *diversity* of the time series. Data generation for simulating changing environments was studied in [16] but no benchmark was developed for these simulations. In effect, they did not base their study on the fundamental structure of time series data and its relationship with time series characteristics. In fact, none of these studies have investigated the presence of differing characteristics in the generation of time series. Instead, we will show that they focus on the creation of either visually similar series or the presence of one particular feature e.g. [14].

**Contribution.** In this paper, we present a methodology to create synthetic time series with the primary aim of ensuring diversity of time series characteristics across the overall dataset. By diversity, it implies that datasets were built to incorporate time series characteristics such as long and short term dependence, non-stationarity, kurtosis, skewness, trend and varying degrees of complexity. This is not the same as introducing uncertainty [17] which would instead mean that the dataset contains time series of various types and behaviors. Diversity is a desirable attribute especially for time series algorithms evaluation purposes, where the algorithms’ performances are required to be evaluated against a wide range of possible situations. We also provide a rigorous validation to measure the degree to which each time series property is contained within the dataset. As part of this work, we generated 53,637 time series to be shared with the time series community [18]. To the best of our knowledge, no other study has constructed this volume of time series *together with a robust evaluation for diversity*. In terms of exploiting this resource, the dataset is provided in full, with evolving documentation to describe its usage together with a link to this paper to provide the researcher with an understanding of the characteristics of subsets of the time series. In terms of data provenance, this paper provides a detailed description of the algorithm used to generate the data. It is also anticipated that by tweaking parameter settings, this repository could grow to more significant number of time series and potentially grow or accelerate research in time series prediction.

**Paper Structure.** The remainder of this paper is organized as follows: in section 2 (The Need for Reliable Time Series Data), we motivate a requirement for this type of method and examine how and why other researchers have created synthetic time series data; in section 3 (Generating Time Series), our method for constructing synthetic time series is presented; in section 4 (A Feature Set to Capture Diversity), we present the fundamental features used to evaluate the synthetic time series; in section 5 (Evaluation), we present our validation together with a detailed discussion of the results; and in the final section, we present our conclusions.

## The need for reliable time series data

Traditional (statistical) applications of time series prediction have been practiced under the assumption that the time series was produced by a linear continuous process [19]. However, this may not be the case where time series are the output of interactions of many alternating series and thus, linearity cannot always be assumed [20]. However, many processes such as financial time series are fundamentally characterized by complex, substantially noisy, dynamic, and nonlinear behaviour [21].

Data generation has been widely used in time series analysis through the use of surrogate data analysis [8], synthetic data generation [7] or simulated data[10]. Surrogate data analysis can be used as a means of estimating the impact of the scale of a characteristic in a time series, through the comparison of the given time series with surrogate series [8]. This can be demonstrated by estimating for example, the impact of non-linearity in a time series in comparison to a series generated from a linear models such as ARIMA and thus, allows researchers to replicate statistical features such as auto-correlation [22].

The majority of practices in time series generation typically use linear approaches, such as the ARIMA family of models. These models establish fundamental statistical consistency, by means of reproducing the mean, variance and auto-correlations of lags of the parent historical data [7]. However, many real-world time series show substantially more complex statistical properties; for example, time series with skewness rather than Gaussian distributions, or those characterized by statistical inter-dependencies [23].

In [7], a Markov chain model was used to generate synthetic data for a wind speed time series analysis. Characteristics such as mean, standard deviation and frequency distribution were predominantly used as assessment metrics. They also evaluated auto-correlation and power spectral density to determined the persistence structure of the series.

In [24], the authors presented a method that incorporates maximum entropy bootstrap to generate ensembles for the given time series data. However, this method only focuses on the low frequency approximation of the signal and discards memory characteristics laid on temporal fluctuations. In [25], the authors also focused on the shape of the signal and tried to use white noise to generate new patterns. This work was originally conducted to compare the performances of time series classification methods on the data for variant representations. However, this work did not address the role of diversity of time series in performance comparisons.

In [26], the authors present a similarity measure that studies generation methods for general time series features. Their work also presents a feature-based time series generation approach that evolves cross-domain time series datasets. The authors present a generic method capable of generating time series from a diversity of domains, as opposed to previous methods that generate time series for particular domains such as weather, economics and energy. This work introduced 4 general attributes for a time series generation method: Dataset-oriented, Deterministic, Stochastic and Innovative. The *Innovative* feature suggests an overlap with our work as it provides a reference to the requirement for *diversity*. However, unlike our approach, their research is bound to domain-specific constraints as it requires examples from the domain for which synthetic time series are to be generated.

In [27], the authors presented a method known as GRATIS and used Mixture AutoRegressive (MAR) models to generate time series data. They incorporated 26 time series features and used a genetic algorithm to evolve time series and create new instances. This approach generated 20,000 yearly, 20,000 quarterly, 40,000 monthly and 10,000 weekly time series based on the MAR models. This work compared their synthetic time series with those of M4 to provide an analysis of coverage and diversity, using M4 as the reference dataset. However, their measures indicate diversity and miscoverage only in relation to the reference dataset after dimension reduction, which is analytically difficult to project into the original feature space. We believe that our approach is free from this limitation.

In [14], a stochastic approach was presented to simulate long range persistence of hydrometeorological time series at multiple scales. The authors use a linear stochastic model to generate synthetic data that replicates the Hurst-Kolmogorov characteristics of the original process. However, this method attempts to replicate temporal dynamics to create similar series, and thus cannot produce diverse series.

More recently, a method called Generative Adversarial Networks (GAN) [15] received attention for generating similar datasets. GANs were originally introduced as an approach that facilitates generative modeling via deep learning. The GANs’ training process is to force the output of the network to follow the distribution of the given input. Most of the studies on GANs focus on image generation and limited work address time series data. Mogren [28] was the first attempt that used GANs to generate continuous sequential data (which is a superset of time series). This work tries to generate new music pieces based on some reference classical musics. A similar attempt was also made in [29], and used GANs to reproduce musical symbolic sequences. Past studies on GANs have also addressed diversity, such as in [30], although in GANs diversity has received a greater attention in the context of training performance, where diversity is required in training samples in order to stabilize modeling performance. More complex practices have also been reported that use deep learning for synthetic data generation. In [31], the authors proposed a deep learning architecture which incorporates a stack of multiple Long-Short-Term-Memory (LSTM) networks and a Mixture Density Network (MDN) for Synthetic Sensor Data Generation. This work attempted to develop a model that reproduces sequences of data which preserve specific statistical properties. However, this work did not consider the diversity of the synthetic data, a characteristic that we believe is crucial when validating results. In fact, none of these studies investigated the presence of differing characteristics when generating synthetic time series. Instead, they focus on the creation of either visually similar series or the presence of one particular feature, as in [14].

In summary, almost all research on surrogate and synthetic time series generation were conducted to reproduce the *same set* of features with *small* variations. To date, there has not been an extensive generation of time series datasets that cover a broad range of time series characteristics and such a method will facilitate a more robust validation of future time series models.

## Generating time series

A time series is a sequence of equally spaced time ordered data points. When conducting time series analysis, the predominant objective is to understand the characteristics of the data, and the extraction of meaningful statistics. Time series data can be broken up into four components [32], [33]: *trend, seasonality, cyclicality*, and *irregularity*.

These components can be used to drive the generation of time series. It is important to note that there is no standard way of combining these components to generate a time series, especially when considering that each component can itself be generated in multiple ways. For instance in [34], the irregularity component is used as the base and manipulated by adding the trend and the seasonality components, which are combined in an additive or multiplicative way.

### Time Series Components

**Trend.**The trend $T_{t}$ describes the long term increase or decrease in the data. The trend can be linear or not, and can be described via the equation in Eq. (1).

(1) $$T_{t}=\left(ax+b\right)\times sin\left(x\right)+cx+d$$

In Eq. (1), symbols a,b,c,d are the coefficients that allow one to specify the desired behavior of the trend, specifically: c,d control the linearity, while a,b via the sinusoidal function determine the non-linear behavior.**Seasonality and Cyclicality.**Both seasonality and cyclicality describe repeating behaviors in the time series data. Specifically, seasonality describes a repeated behavior that occurs at regular intervals (e.g. every number of seconds, days, weeks, etc.); cyclicality, on the other hand, describes repeated behaviors that occurs at irregular intervals. We used four functional forms of repeating patterns to simulate seasonality $S_{t}$: the sinusoidal function, the step-wise function, the impulsive function and the triangular function.

The *Sinusoidal function* is simulated using Eq. (2), where, $\beta_{0}$ and $\beta_{1}$ are the weights; $\alpha_{0}$ and $\alpha_{1}$ are the phases for the sinusoidal functions; and $\alpha_{0},\alpha_{1},\beta_{0}$ and $\beta_{1}$ are constants.

(2) $$S_{t}=\beta_{0}\sin\left(\alpha_{0}t\right)+\beta_{1}\sin\left(\alpha_{1}t\right)$$

The *Step-wise* function is a type of latch function (with two stable states) that changes between two values at fixed intervals, shown in Eq. (3), where, p (p≥0) is the period, t is time and m is an integer.

(3) $$S_{t}=\left\{ \begin{array}{cc} 1 & 2mp<t<2mp+1\\ 0 & 2mp+1<t<2mp+2 \end{array}\right.$$

**Fig. 1:**
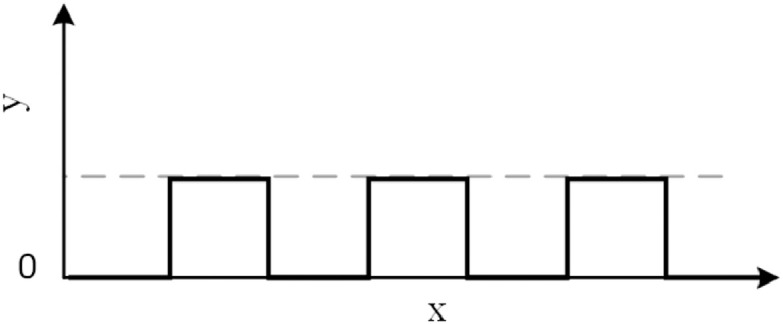
The Step-wise function.

The step-wise function is shown in Fig. 1.

The **Triangular** function is similar to the step-wise function where the step-wise effect occurs in the slope of the line. Eq. (4) implements the triangular function with a fixed slope α, with $b_{0}$ and $b_{1}$ as constants, p as the period, t as time, and m as an integer.

(4) $$y=\left\{ \begin{array}{cc} \alpha t+b_{0} & 2mp<t<2mp+1\\ -\alpha t+b_{1} & 2mp+1<t<2mp+2 \end{array}\right.$$

**Fig. 2:**
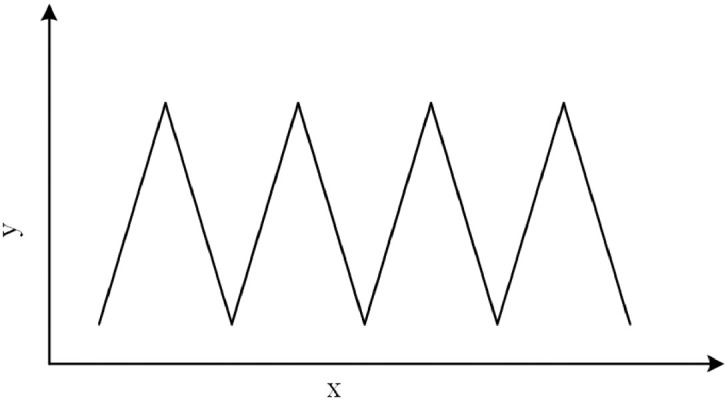
The Triangular function.

The Triangular function is shown in Fig. 2.

The *Impulsive function* is a pattern that has a value of 1 at fixed intervals and 0 otherwise and was implemented using (5), where t is time, and p is the period.

(5) $$y=\left\{ \begin{array}{cc} 1 & \left\lfloor t/p\right\rfloor =t/p\\ 0 & otherwise \end{array}\right.$$

**Fig. 3:**
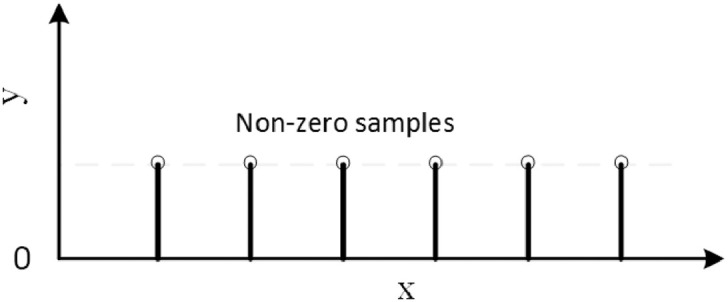
The Impulsive function.

The Impulsive function is shown in Fig. 3.

Note that the impulsive function is smoothed using a moving average operation to avoid sharp fall and rise fluctuations.**Irregularity.**The Irregular component describes behaviors that cannot be represented via the trend or the cyclicality/seasonality. In time series analysis this component is often referred to as *noise*. Some researchers, e.g. [35], believe that this component carries important information. Therefore, in order to accommodate for these theories, we model the noise as a signal with its own characteristics. Specifically, we consider following three models:

• Fractional Gaussian noise (fGn), which represents stationary series with a constant mean and variance;
• Fractional Brownian motions (fBm), which are non-stationary series with time-dependent variance [36];
• Multi-fractal Brownian motion, for the case where the Hürst exponent is applicable to time series, that is: the index is a function of time.

The Hurst exponent H [37] is one of the most popular methods to measure Long Range Dependence (LRD). H attempts to explain LRD as a property of stochastic self-similar processes. Here, x(t) is self-similar with the Hurst exponent H, when for a stretching factor λ, the rescaled process x(λt) is equal to the original process x(t) in terms of distribution as in Eq. (6), where ≐ denotes *equality* in terms of distributions.

(6) $$x\left(t\right)≐\lambda^{-H}x\left(\lambda t\right)$$

If the fluctuations are stationary (the process has a constant mean and a constant variance), the process is said to have fractional Brownian motion (fBm). Based on [38], the auto-correlation function for fBm processes is defined in Eq. (7).

(7) $$\rho \left(k\right)=\frac{1}{2}\left({\left|k+1\right|}^{2H}-2{\left|k\right|}^{2H}+{\left|k-1\right|}^{2H}\right)$$

Based on [37], applying a first-order Taylor expansion to ρ(k) from Eq. (7) delivers the functionality in Eq. (8), for k→∞.

(8) $$\frac{\rho (k)}{{H\left(2H-1\right)|k|}^{2H-2}}\to 1$$

It can be inferred from Eq. (8) that the autocorrelation $\rho \left(k\right)\propto {\left|k\right|}^{2-2H}$ when $H>\frac{1}{2}$, based on [37].

### Our Combinational methodology

Building on the work presented in [39], we combine *trend* and *cyclicality* into a joint component known as the *trend-cycle* component in order to prevent known complexities involved in identifying *Cyclicality*. In our time series construction method, we consider all possible additive and multiplicative combinations of *trend-cycle* $T_{t}^{c}$, *seasonality* $S_{t}$ and *irregularity* $I_{t}$, using the approach presented in [40], where there are 8 possible models for combining $T_{t}^{c}$, $S_{t}$ and $I_{t}$, shown in Table 2.

**Table 2:** Time Series Component Combinations

Model	Description
Model 1	$Y_{t}=T_{t}^{c}+S_{t}+I_{t}$
Model 2	$Y_{t}=\left(T_{t}^{c}+S_{t}\right)I_{t}$
Model 3	$Y_{t}=\left(T_{t}^{c}+I_{t}\right)S_{t}$
Model 4	$Y_{t}=\left(S_{t}+I_{t}\right)T_{t}^{c}$
Model 5	$Y_{t}=T_{t}^{c}S_{t}+I_{t}$
Model 6	$Y_{t}=T_{t}^{c}I_{t}+S_{t}$
Model 7	$Y_{t}=S_{t}I_{t}+T_{t}^{c}$
Model 8	$Y_{t}=T_{t}^{c}S_{t}I_{t}$

In Table 2, Model 1 is the pure additive model which is the most widely used model in the time series community. Model 8, or the pure multiplicative model, is the second most popular model among time series researchers. The other models in Table 2 are also used in the time series studies with *Model 3* and *Model 5* being more popular because they incorporate irregularity $I_{t}$ using an addition operation.

**Fig. 4:**
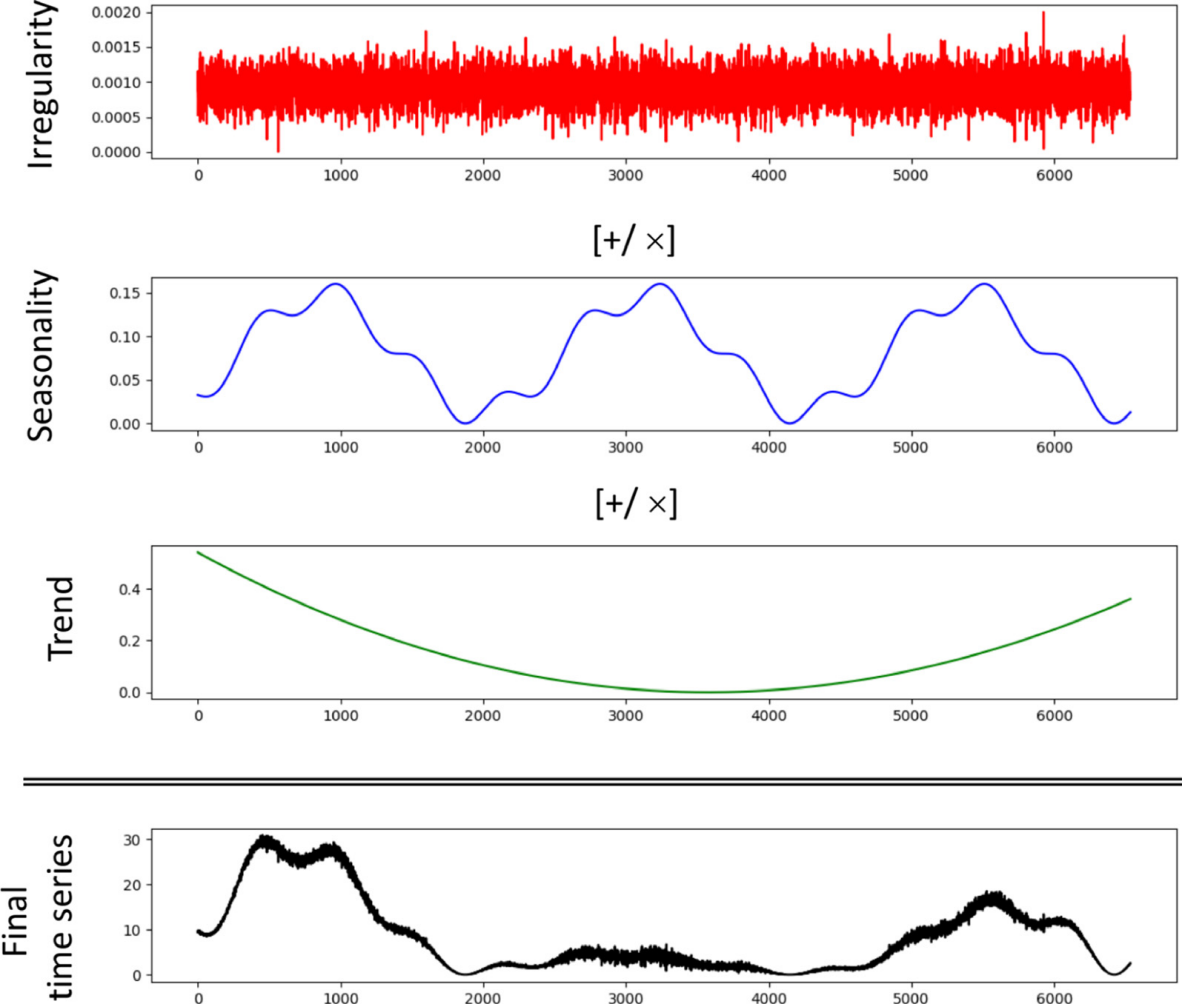
Combining Time Series components.

An example of combining time series components is illustrated in Fig. 4.

## A feature set to capture diversity

In the previous section, we described a method that ensures diversity in synthetic time series using different combinations of functions that introduce time series characteristics. As one cannot directly measure time series components such as trend, it is necessary to extract a feature set to support any evaluation. For our validation framework in this paper, we use *Long-Range Dependence, Complexity* and *Normality*.

### LRD

Long-Range Dependence (LRD) measures the degree of dependence (correlation) over long intervals of time, which is a way to indicate “memory” in a time series. As mentioned earlier, the Hurst exponent is the traditional method for measuring LRD [41]. The Hurst exponent divides time series data into three categories: Negatively-correlated 0<α<0.5, Uncorrelated α≃0.5 and Correlated 0.5<α<1. However, the Hurst exponent is only able to process *stationary* time series. An alternative to the Hurst exponent for measuring LRD is the Detrended Fluctuation Analysis (DFA) approach which allows for the detection of LRD in *non-stationary* time series.

Using Detrended Fluctuation Analysis (DFA), LRD can be assessed and categorized into 6 well known and critical classes.

Values of α can be interpreted as follows: α=1 indicates perfect (self) similarity in the data (a characteristic of the Self Organized Critically systems [42]); α=1/2 represents white noise, no similarity (or no memory); 1/2<α<1 describes positive correlation, with similarity (memory) increasing with the values of α; α<1/2 indicates inverse correlation; α>1 indicates that while correlations exist, they cannot be described in the form of a power-law relationship. A special case where α=1.5, indicates Brownian noise or the integration of white noise. α also provides information about the roughness of the time series where larger values of α belong to smoother time series. 1/f noise can be interpreted as a compromise between the complete unpredictability of white noise (very rough landscape) and the very smooth landscape of Brownian noise.

### Complexity

Entropy can be used to measure complexity [43]. As per [44], given a signal y with sample size N and tolerance r, sample entropy is the negative logarithm of the conditional probability that a sub-series of length m matches point-wise with the next point with tolerance (distance less than) r. In this paper, we used *spectral* entropy to evaluate complexity, which measures the uniformity of the power spectrum distribution or the frequency component distribution.

### Normality

Normality is a test to determine if data falls into a normal distribution [45]. Common metrics to measure normality are: Kurtosis, Skewness, and Gaussianity of the Differences (GoD). Kurtosis measures the number of outliers in the dataset with respect to a normal distribution: when Kurtosis is high, the dataset has a higher number of outliers (heavy tail in the distribution); when kurtosis is low, the outliers are low to none (light tail). Skewness measures the symmetry of the distribution: when positive, the distribution has a longer or fatter tail on the right side; when negative, the left side of the distribution has a longer or fatter tail; when zero, the distribution is symmetrical. Gaussianity of the differences (GoD) was used in [33], [46] to measure the normality of the distribution of the changes (the first difference of the series) in the time series: this is an important metric because differencing is an important phase of many time series analysis.

## Evaluation

As the goal of this research is to provide researchers with a method to create *diverse* time series datasets, it must be accompanied by validation framework to *measure* diversity. There are five metrics used to assess the time series components discussed in the previous section : Detrended Fluctuation Analysis (DFA), Spectral Entropy, Kurtosis, Skewness, and Gaussianity of the differenced values. They have been extensively used in the literature as individual assessment measures of time series data [10].

### Evaluation criteria

In this paper, the evaluation goal is to demonstrate the diversity of the generated time series, where the goal is to achieve maximum diversity and thus, we incorporate three main approaches to assess the degree of diversity. First, we use the histogram plot to visually observe the diversity of the generated time series for each feature on an individual basis. In a histogram plot, the x-axis represents the range of values for a given feature and the y-axis shows the number of time series that fall into each specific interval. Therefore, using the histogram plot, we can visually observe the distribution of the time series for each feature, individually. Note that we are not looking for a histogram with a uniform distribution of values but instead, attempt to determine the non-empty intervals, so that we obtain time series for *all* feature values.

Second, we use the multivariate entropy score to provide an accumulative diversity score across individual features to return a single score for diversity. While it represents the dominant approach in the literature, the problem with the multivariate entropy score is that it calculates diversity independent of inter-feature relationships. Therefore, we propose the third evaluation measure known as the *coverage rate* which provides a more reliable evaluation for diversity.

### Visualizing feature metrics

**Fig. 5:**
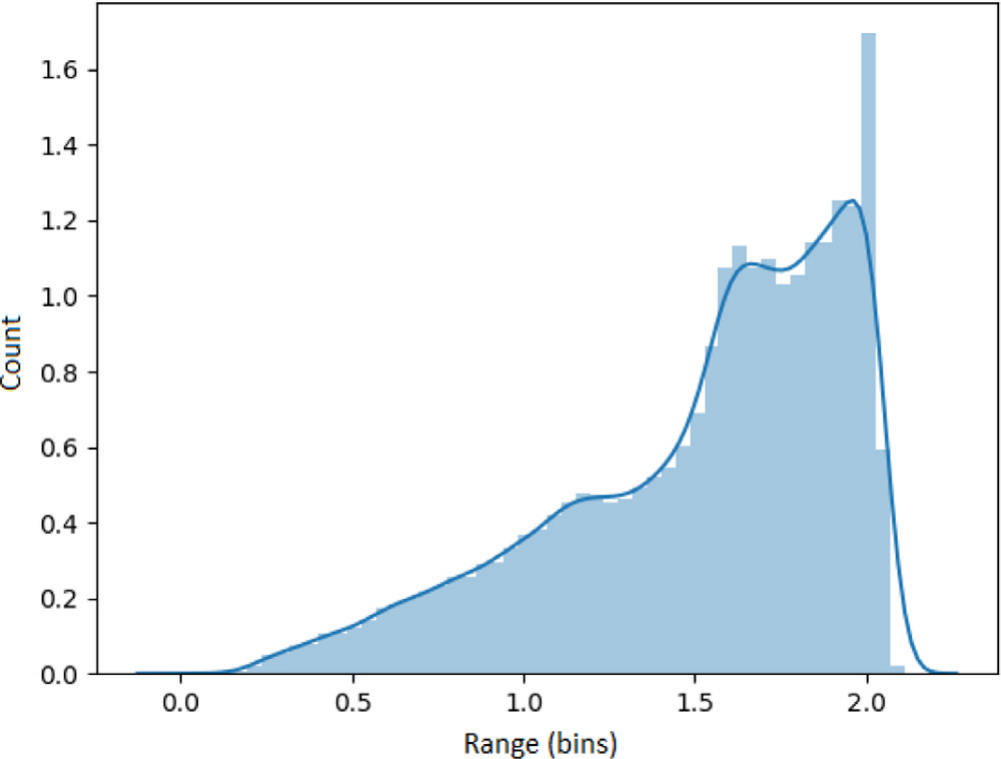
Results for LRD Metric (DFA).

**Fig. 6:**
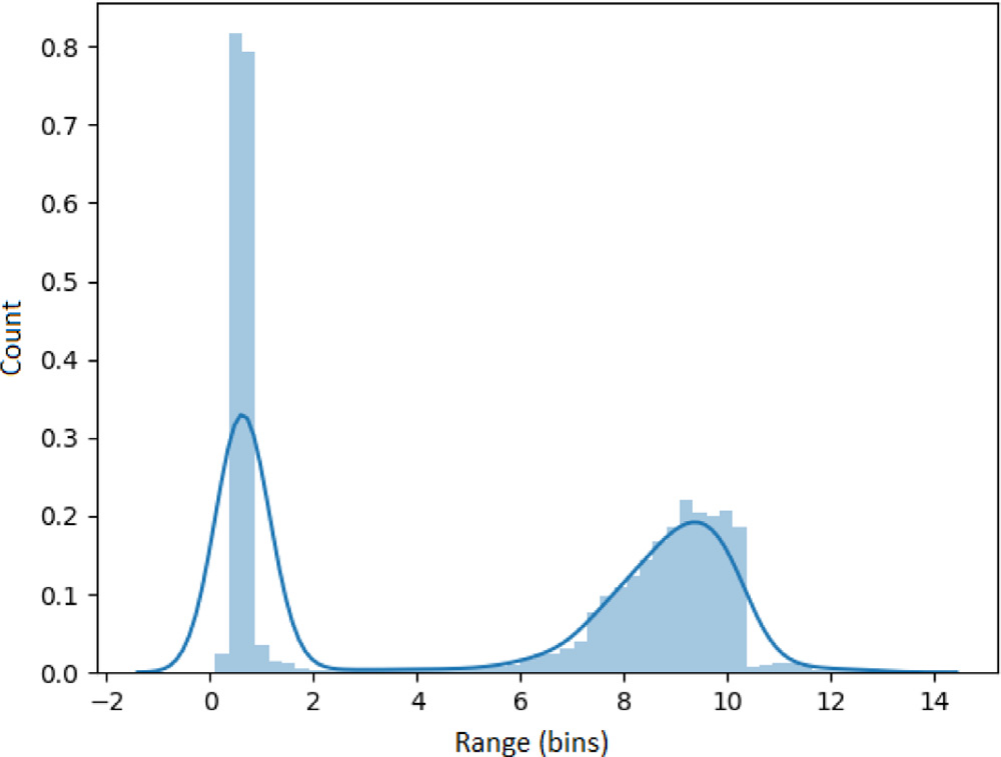
Results for Complexity.

**Fig. 7:**
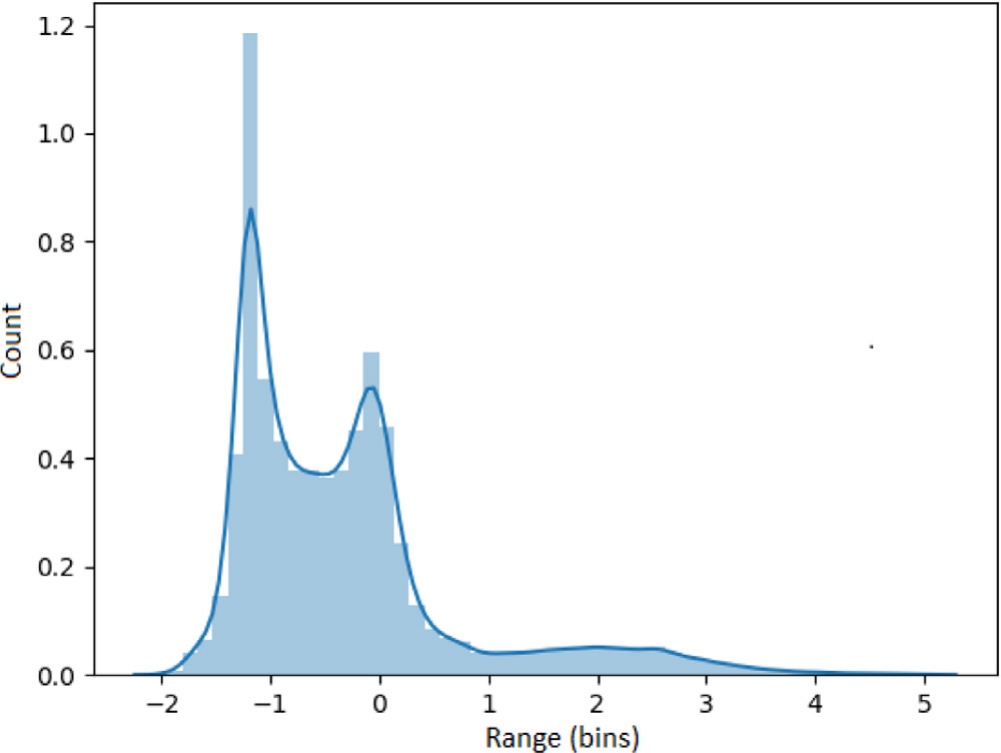
Results for Normality (Kurtosis).

**Fig. 8:**
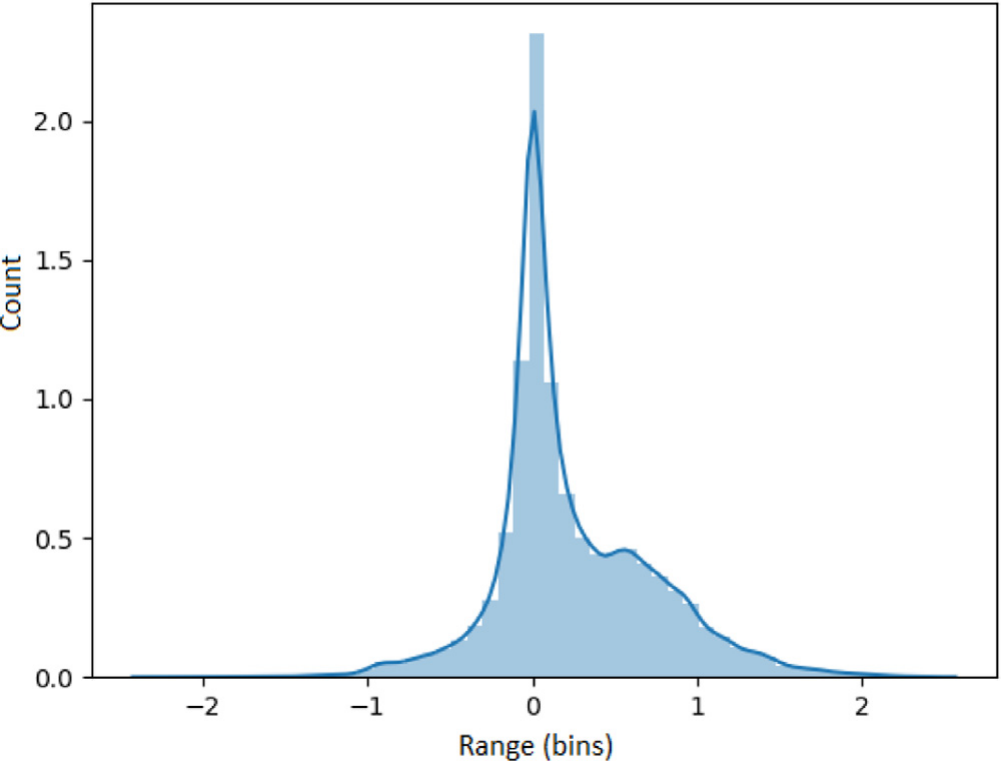
Results for Normality (Skewness).

**Fig. 9:**
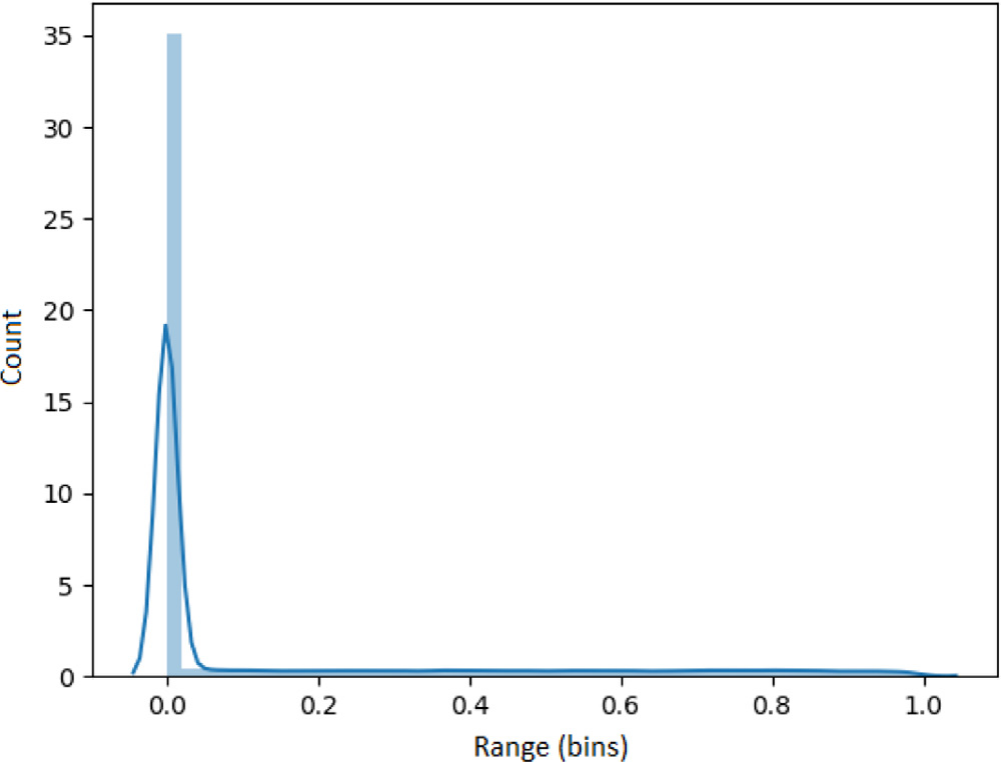
Results for Normality (GoD).

The results presented in this section use the 53,637 generated time series as input. Each set of results presented in Fig. 5, Fig. 6, Fig. 7, Fig. 8, Fig. 9 represents an analysis (metric) of a specific feature across the entire dataset. For each feature, a histogram plot has been provided that visually illustrates the diversity of time series over the potential range of values for the corresponding feature. Using these histograms, we evaluate the diversity of each feature independent of each of the other features and this evaluation can be referred to as *feature specific* diversity. As the goal is to observe the least number of empty intervals, a perfectly diverse dataset is one that has no zero intervals over the range of the possible values for the given feature.

Long range dependency for each series was calculated using a DFA analysis and shown in Fig. 5. These results demonstrate that the synthetic series encapsulate all forms of long range dependency described by the DFA values in the previous section.

In Fig. 6, the histogram for spectral entropy of the time series is presented. The entropy of values close to zero indicates high levels of self-similarity and thus, higher predictability. The results illustrate a high number of time series with low complexity (entropy close to zero) and also a high number of complex time series (entropy greater than 9). In addition, there are a lot of time series between these ranges, demonstrating that *all* complexity levels are present.

In Fig. 7, the results for kurtosis show the expected diversity of negative (series of light tails or series of no outliers), zero (occasional outliers) and positive values (series of heavy tails or series with significant or numerous outliers). This is a strong indicator of diversity across the datasets.

The results for skewness are presented in Fig. 8, showing a high number of time series with negative skewness (series with a fatter or longer tails on the left side), zero skewness (series of symmetrical distribution) and positive skewness (series of heavy or long tails on the right side). Once again, this indicates a high level of diversity across the datasets.

Fig. 9 illustrates the distribution of the gaussianity of the differences. A value of 1 indicates that the series follow a normal/Gaussian distribution and a value of 0 indicates no normality. The results show the generated series cover the entire range between zero and complete normality and thus, demonstrates a high level of diversity for the generated series.

### Multivariate entropy score

The diversity measure in this paper is based on Shannon’s entropy function which is frequently used to measure the amount of information in an encoded message [47], and shown in Eq. (9), where $x_{1},x_{2},...,x_{S}$ are the possible values of X and $p(x_{i})$ is the probability of observing $x_{i}$ or $X=x_{i}$.

(9) $$H\left(X\right)=-\sum_{i=1}^{S}p\left(x_{i}\right)\log p\left(x_{i}\right)$$

In order to measure diversity, we used a metric known as the *evenness* measure [47], which provides a normalized value for H(X) based on its maximum, and shown in Eq. (10).

(10) $$H_{max}\left(X\right)=-\sum_{i=1}^{S}\frac{1}{S}\log\frac{1}{S}=\log S$$

Therefore, the *diversity* of feature X is calculated by Eq. (11).

(11) $$H_{E}\left(X\right)=\frac{H(X)}{H_{max}\left(X\right)}=-\frac{1}{\log S}\sum_{i=1}^{S}p\left(x_{i}\right)\log p\left(x_{i}\right)$$

In this evaluation, we assume that all the features have equal significance, independent of the domain-specific constraints of the problem space. Assuming that all features have the same significance, the diversity for a multivariate (multi-feature) dataset with k features can be obtained using Eq. (12), where H will range between 0 and 1.

(12) $$H=\frac{1}{k}\sum_{i=1}^{k}H_{E}\left(X^{k}\right)$$

In order to implement this metric, each feature was categorized into buckets/zones as used traditionally by researchers. The categorization of the features, later shown in Table 3, is as follows:

• Spectral Entropy was categorized into three categories including A:X<1, B:1≤X<9 and C:9≤X.
• Kurtosis was categorized into three categories including A:X<−0.3, B:−0.3≤X<0.3 and C:0.3≤X.
• Skewness was categorized into three categories including A:X<−0.3, B:−0.3≤X<0.3 and C:0.3≤X.
• GoD was categorized into two categories including A:X<0.02 and B:0.02≤X.
• DFA was categorized into seven categories including A:X<0.45, B:0.45≤X<0.55, C:0.55≤X<0.95, D:0.95≤X<1.05, E:1.05≤X<1.45, F:1.45≤X<1.55, G:1.55≤X.

**Table 3:** Proportion of dataset relative to time series characteristics.

Feature	A	B	C	D	E	F	G
Spectral Entropy	0.42	0.28	0.29	N/A	N/A	N/A	N/A
Kurtosis	0.59	0.25	0.15	N/A	N/A	N/A	N/A
Skewness	0.07	0.58	0.35	N/A	N/A	N/A	N/A
GoD	0.70	0.3	N/A	N/A	N/A	N/A	N/A
DFA	0.017	0.012	0.092	0.035	0.190	0.067	0.584

Table 3 shows the breakdown of the proportion of series that belong to each of the categories outlined above. An N/A implies that this category is not appropriate for that metric.

**Fig. 10:**
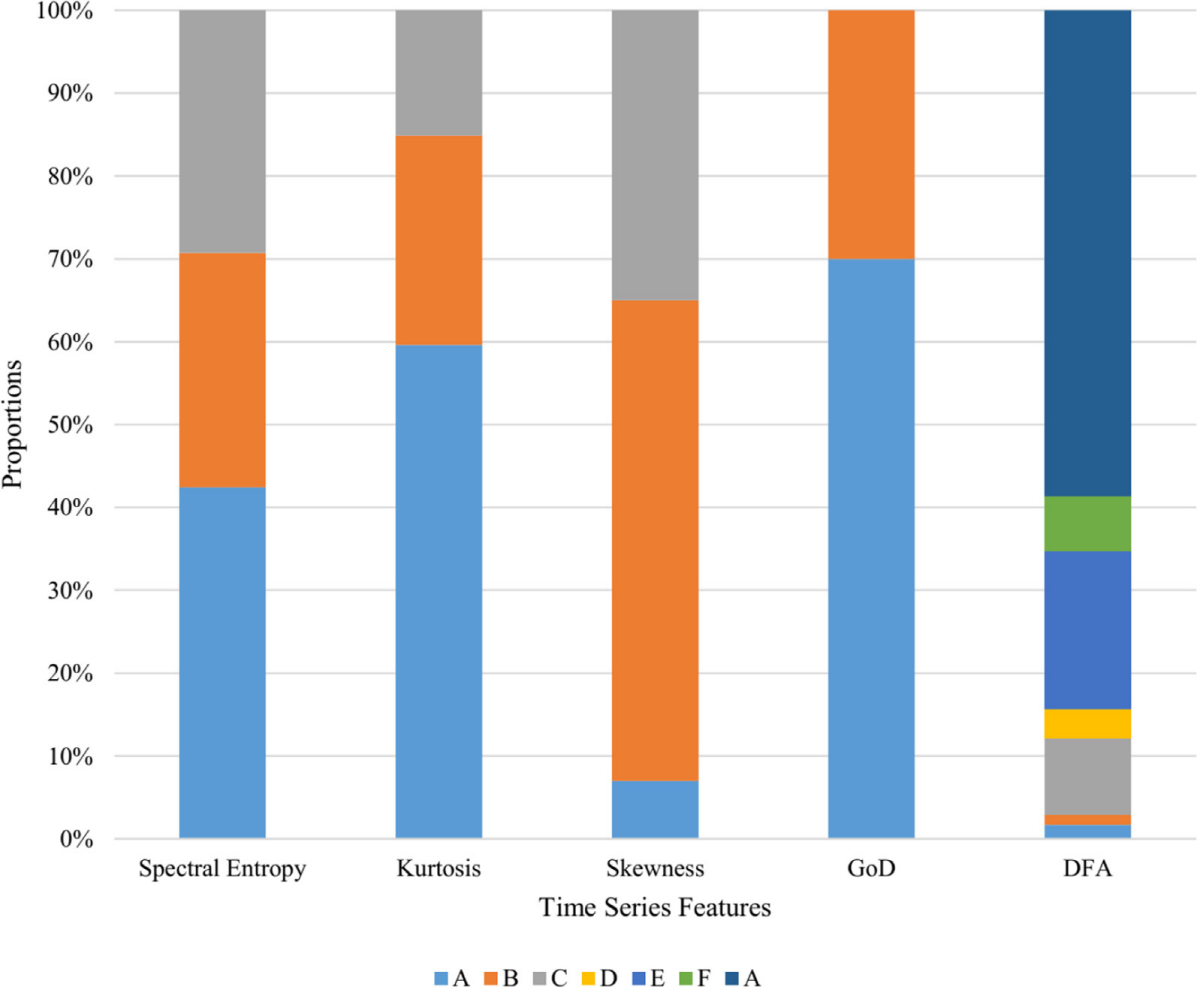
Proportion of dataset relative to time series characteristics.

The proportion of series that belong to each of the categories outlined above are shown in Fig. 10

Table 4 shows the $H_{max}$ and $H_{E}$ of each metric for the full dataset. These interim results are used to calculate the diversity as our final evaluation is to measure the diversity and coverage rate. The overall diversity score, H for the dataset was 0.83. Here, H(X), $H_{max}$ and $H_{E}$ which were obtained using Eqs. (9), (10) and (11), and show that the level of diversity for each of the metrics examined ranges between 0.65 for DFA to 0.98 for Spectral Entropy. This is a significant result as it indicates the most diverse features or the features that have the best evenness. The low level of diversity for the DFA metric was predominantly due to the low levels of stationary data DFA<1.

**Table 4:** H scores for each metric.

Feature	H(X)	$H_{\max}$	$H_{E}$
Spectral Entropy	1.55	1.58	0.98
Kurtosis	1.366	1.58	0.86
Skewness	1.25	1.58	0.79
GoD	0.88	1.00	0.88
DFA	1.837	2.80	0.65

**Fig. 11:**
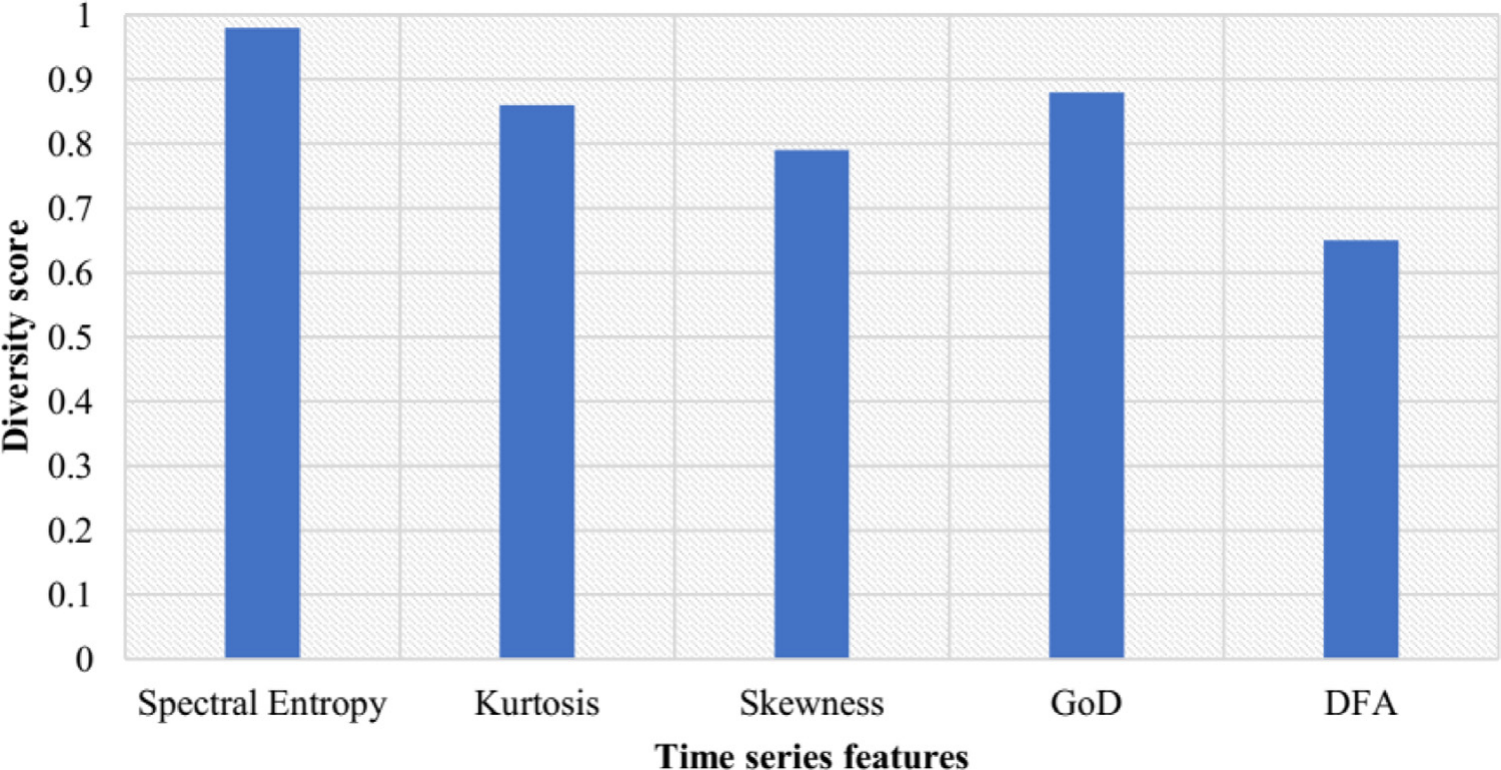
H scores for each metric.

The H score (diversity score) for each time series characteristics is shown in Fig. 11

### Feature space coverage

The feature space for the data is identified as all potential category combinations of the metrics outlined above. For this evaluation, we selected a measure of diversity that reflects the *percentage coverage* of the samples over the *potential* feature space. Using Table 3, there are: 3 categories for spectral entropy; 3 categories for Kurtosis; 3 categories for Skewness; 2 categories for GoD; and 7 categories for DFA. Thus, there is a total of (3×3×3×2×7) 378 possible feature combinations, meaning 378 potential categories from our metrics. A full list of all feature combinations is provided in [18].

**Fig. 12:**
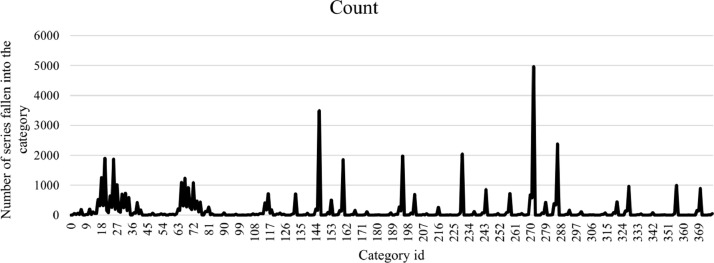
Number of series in each category.

Figure 12 shows the number of time series where a specific category was represented by our synthetic data. Here, the *x-axis* represents all 378 possible categories, each using a unique category_id, and the *y-axis* shows the number of time series that fall under that category_id. For some categories, it is clear that there are multiple time series in the datasets whereas other categories are absent altogether.

## Conclusions

Researchers using time series data are often faced with the problem of insufficient data for the purposes of testing and validating their algorithms. In this work, we presented a methodology for the creation of a large number (53,637) of time series which are now available to the research community [18]. Their construction had an emphasis on *diversity* and a validation framework to ensure a robust evaluation of the synthetic time series created. Our method comprised 5 well-known time series features and used a multivariate entropy measure to examine the diversity of the created time series based on these five features. The experimental results showed that our overall dataset measured diversity at 83.4%, which we believe to be a significant achievement. We have also proposed a new diversity assessment measure called the *coverage rate* which reflects the coverage of the dataset over the full feature space. The results show that our series exhibit a coverage rate of 72%, which delivers a significant contribution for such a large dataset.

There are some limitations to this research which we feel should be highlighted. Firstly, our paper considers only five features for studying diversity and future research could adopt more time series features into a more advanced study of diversity when building synthetic time series. Additionally, this research constructs only 50K time series and a wider set would be necessary to accommodate the additional features. Secondly, we assume that all features have equal significance, independent of the domain-specific constraints of the problem space. To advance our work, researchers could incorporate an additional customization step for determining the significance of features, applicable to *each* specific domain.

## Declaration of the Conflict of Interest

The authors declare that there are no known competing financial interests or personal relationships which could have appeared to affect the research presented in this paper.
